# Antibody response following the third and fourth SARS-CoV-2 vaccine dose in individuals with common variable immunodeficiency

**DOI:** 10.3389/fimmu.2022.934476

**Published:** 2022-07-28

**Authors:** Bibi Uhre Nielsen, Camilla Heldbjerg Drabe, Mike Bogetofte Barnkob, Isik Somuncu Johansen, Anne Kirstine Kronborg Hansen, Anna Christine Nilsson, Line Dahlerup Rasmussen

**Affiliations:** ^1^ Department of Infectious Diseases, Copenhagen University Hospital, Rigshospitalet, Copenhagen, Denmark; ^2^ Department of Clinical Immunology, Odense University Hospital, Odense, Denmark; ^3^ Department of Infectious Diseases, Odense University Hospital, & Research Unit for Infectious Diseases, University of Southern Denmark, Odense, Denmark; ^4^ OPEN, Odense Patient data Explorative Network, Odense University Hospital, Odense, Denmark

**Keywords:** cvid, sars-cov2, covid-19, corona vaccination, booster doses

## Abstract

**Background:**

The antibody response after vaccination is impaired in common variable immunodeficiency (CVID).

**Objective:**

We aimed to study the spike receptor-binding domain IgG antibody (anti-S-RBD) levels during a four-dose SARS-CoV-2 vaccination strategy and after monoclonal antibody (mAB) treatment in CVID. Moreover, we assessed the anti-S-RBD levels in immunoglobulin replacement therapy (IgRT) products.

**Methods:**

In an observational study, we examined anti-S-RBD levels after the second, third, and fourth dose of mRNA SARS-CoV-2 vaccines. Moreover, we measured anti-S-RBD after treatment with mAB. Finally, anti-S-RBD was assessed in common IgRT products. Antibody non-responders (anti-S-RBD < 7.1) were compared by McNemar’s test and anti-S-RBD levels were compared with paired and non-paired Wilcoxon signed rank tests as well as Kruskal–Wallis tests.

**Results:**

Among 33 individuals with CVID, anti-S-RBD levels increased after the third vaccine dose (165 BAU/ml [95% confidence interval: 85; 2280 BAU/ml], *p* = 0.006) and tended to increase after the fourth dose (193 BAU/ml, [−22; 569 BAU/ml], *p* = 0.080) compared to the previous dose. With increasing number of vaccinations, the proportion of patients who seroconverted (anti-S-RBD ≥ 7.1) increased non-significantly. mAB treatment resulted in a large increase in anti-S-RBD and a higher median level than gained after the fourth dose of vaccine (*p* = 0.009). IgRT products had varying concentrations of anti-S-RBD (*p* < 0.001), but none of the products seemed to affect the overall antibody levels (*p* = 0.460).

**Conclusion:**

Multiple SARS-CoV-2 vaccine doses in CVID seem to provide additional protection, as antibody levels increased after the third and fourth vaccine dose. However, anti-S-RBD levels from mAB outperform the levels mounted after vaccination.

**Clinical Implications:**

Boosting with SARS-CoV-2 vaccines seems to improve the antibody response in CVID patients.

**Capsule summary:**

The third and possibly also the fourth dose of mRNA SARS-CoV-2 vaccine in CVID improve the antibody response as well as stimulate seroconversion in most non-responders.

## Introduction

Severe acute respiratory syndrome coronavirus-2 (SARS-CoV-2) has already caused coronavirus disease (COVID-19) in half a billion people and caused 6 million deaths worldwide (1). Vaccines against SARS-CoV-2 have been rapidly developed and implemented globally in an attempt to combat the ongoing pandemic by limiting the viral spread and preventing severe illness. The vaccine-induced antibody response has been shown to correlate with protection against severe disease in immune-competent individuals (2). However, in individuals with primary immune deficiency (PID), we have little knowledge about the effectiveness and the optimal vaccination strategy.

Common variable immunodeficiency (CVID) is the most common symptomatic PID among adults, with the Danish prevalence estimated to be 1:26,000 (3). CVID is a primary antibody deficiency characterized by hypogammaglobulinemia, a reduced frequency of isotype switched memory B cells and/or a poor vaccination response (~insufficient production of specific antibodies), which leads to frequent respiratory infections. Most CVID patients receive immunoglobulin replacement therapy (IgRT) from healthy donors to supplement the deficient antibody production (~passive immunization). The concentration of SARS-CoV-2-specific antibodies in the current IgRT preparations are unknown, but recent publications have indicated increasing amounts (4, 5). Still, it is not known which levels are needed to confer protection against SARS-CoV-2 infection. As such, CVID patients need other protective measures against infection.

A recent study has demonstrated that it is safe to vaccinate CVID patients with mRNA-based vaccines (6). However, the proportion of CVID patients who develop specific antibodies after the second vaccine dose have varied between cohorts, ranging from 20% to 80% (7–10). Although the level of immunity, including both T- and B-cell response, are lower in CVID patients than that of healthy controls after two vaccine doses (11), CVID patients may still benefit from SARS-CoV-2 vaccination (10).

In Denmark, the SARS-CoV-2 vaccination strategy was based on risk assessment for severe outcomes of COVID-19. As the risk for severe COVID-19 was assumed to be high for CVID patients (12), CVID patients in Denmark have been offered early vaccination and boosting with a total of four doses of mRNA-based SARS-CoV-2 vaccines over a period of 16 months. However, the four-dose strategy has not been evaluated in people with CVID and the antibody response has therefore not been elucidated.

Based on studies showing clinical efficacy of treatment with SARS-CoV-2 monoclonal antibodies (mAB), high-risk immunocompromised patients including CVID patients have been offered this therapy post-infection (13). We hypothesize that compared to vaccines, pre-exposure prophylaxis with mAB could potentially provide better protection against severe COVID-19 in some CVID patients.

Herein, we assessed the dynamics of SARS-CoV-2 antibody levels during a four-dose SARS-CoV-2 vaccination program and after treatment with mAB, to compare the outcome of vaccination and passive immunization in CVID. Moreover, we examined the levels of antibodies in common IgRT products.

## Methods

This observational, retrospective, single-center study, investigated SARS-CoV-2 antibody levels in a Danish CVID cohort. Approximately 230–250 individuals with CVID live in Denmark and one-fifth are followed for care at the Department of Infectious Diseases at Odense University Hospital (OUH), Southern Denmark. The antibody response of CVID patients was monitored at regular hospital consultations after primary SARS-CoV-2 vaccination (second dose) and after a third and fourth dose. When mAB therapy (sotrovimab and REGN-COV2) became available in Denmark in November 2021, we initiated monitoring of antibody levels before and after this treatment. mAB was offered to CVID patients after considering age, clinical severity of CVID, and antibody levels. During the study, SARS-CoV-2 antibody levels were also assessed in samples of IgRT products.

### Population

We included all CVID patients at OUH, 18 years or older, who consented to the study and had at least one measurement of SARS-CoV-2 antibodies performed. CVID was defined according to the revised (2014) European Society for Immunodeficiency (ESID) diagnostic criteria for CVID (14, 15). Some patients diagnosed prior to 2014 had normal CD4 cell count but naïve T cells <10%. In these patients, T-cell proliferation has not been analyzed, as normal T-cell proliferation was first added as a criterion in 2014. Therefore, these patients only met the old ESID criteria (16) for probable CVID, but they were still included as CVID in this study. Medical records were used to obtain information about CVID, oral glucocorticoid use, previous CD20 inhibitors, IgRT, SARS-CoV-2 vaccination, COVID-19, and treatment with mAB.

### Samples

In SARS-CoV-2 vaccinated patients, serum samples were drawn 3 (± 0.5) months after the second and third vaccine dose and 1 (± 0.5) month after the third and fourth dose. Furthermore, blood was collected 1 (± 0.5) month after mAB was administered to patients with COVID-19. COVID-19 was defined as a positive real-time polymerase chain reaction (RT-PCR). IgRT samples were remainders from common preparations used to treat CVID. The types of IgRT included were Privigen and Octagam (intravenous treatment) and Hizentra and HyQvia (subcutaneous treatment). IgRT samples were tested as patient samples, except for Hizentra preparations, which were diluted 1:2 with human albumin 5% prior to testing. All reported results were normalized to a concentration of 100 mg immunoglobulin/ml to remove bias related to IgG concentration in different preparations.

### Serologic analysis of SARS-CoV-2 antibodies

SARS-CoV-2 spike protein receptor-binding domain IgG antibodies (anti-S-RBD) was determined using the SARS-CoV-2 IgG II Quant assay (Abbott Laboratories). This assay is calibrated against the first WHO International Standard for anti-SARS-CoV-2 immunoglobulin (NIBSC code 20/136) (17), enabling issuing of immunogenicity results in standardized units; binding antibody units (BAU)/ml. Abbott arbitrary unit (AU) is converted to BAU with the following equation: 1 BAU/ml = 0.142 × AU/ml (18). In accordance with the manufacturer’s instructions, levels ≥7.1 BAU/ml were considered positive and defined as responders and levels <7.1 BAU/ml were defined as non-responders. Antibody levels were divided into four categories (<7.1, 7.1–205, 206–817, and >817 BAU/ml). The cutoffs correspond to the mid (205 BAU/ml) and the high (817 BAU/ml) geometric mean of anti-S-RBD titers after natural infection in the WHO reference panel (19).

SARS-CoV-2 nucleocapsid IgG antibodies (anti-N) were determined using the qualitative SARS-CoV-2 IgG assay (Abbott Laboratories) to detect patients who were convalescent after COVID-19.

### Statistical analysis

Data were reported as proportions for categorical variables and medians with interquartile ranges (IQR) for continuous variables. After excluding patients who received mAB, the proportions of patients who did not seroconvert (anti-S-RBD <7.1 BAU/ml) vs. those who seroconverted (anti-S-RBD ≥7.1 BAU/ml) were compared using McNemar’s test. Paired Wilcoxon signed rank tests were used to compare anti-S-RBD levels between different SARS-CoV-2 vaccine doses and before vs. after mAB. Non-paired Wilcoxon signed rank test was used to compare anti-S-RBD levels in those vaccinated with four doses to those receiving mAB without having the fourth dose. Kruskal–Wallis test was used to test for variation in antibody concentrations in different IgRT products. Furthermore, Kruskal–Wallis tests were used to compare patient anti-S-RBD levels by IgRT treatment, including no treatment, at different vaccine time points. Kruskal–Wallis tests were also used in sensitivity analyses to compare anti-S-RBD (1) in those with and without oral glucocorticoid therapy, (2) in those with and without previous CD20 inhibitors, and (3) in those presenting with infections only vs. those presenting with autoimmune/granulomatous/lymphoproliferative disease. In Wilcoxon signed rank test, we compared anti-S-RBD levels in those meeting only the old ESID criteria to those who meet the revised criteria after the second dose. All statistical tests were considered significant at *p* < 0.05. All data were registered in a REDCap database hosted by OPEN (20) and statistical analyses were performed using R (version 4.1.1) and RStudio (version 1.4.1106).

### Ethical considerations

The study was approved by The Danish Data Protection Agency (Journal nr. 21/37904). Oral and written informed consent for the publication of data included in this article were obtained from all patients. The study did not require ethical approval as the blood samples were collected as a standard of care.

## Results

In total, 52 patients followed for CVID in our outpatient department were invited to participate in the study. Forty-two consented and 33 were included (**Supplementary Figure 1**). Of these, 22 (67%) fulfilled the revised ESID criteria for CVID, while 11 (33%) had a normal CD4 cell count but a naïve T-cell fraction <10% and no examination of T-cell proliferation. The median age was 50 years (IQR: 32; 68), 42% were men, and 85% received IgRT. Five patients (15%) were treated with low-dose oral glucocorticoids (≤7.5 mg/day) and 3 (9%) had received CD20 inhibitors after the CVID diagnosis. During the study, 85% received three doses and 48% of the patients received all four doses of the SARS-CoV-2 vaccine. All but one of the vaccinated CVID patients received all vaccine doses with Pfizer/BioNTech (Comirnaty), whereas the remaining patient received all doses with Moderna (Spikevax). Three unvaccinated patients were all diagnosed with COVID-19. One of the unvaccinated patients were infected twice, first with an unknown variant (October 2020) and later with a Delta variant (November 2021). A total of 19 (58%) of the vaccinated patients developed breakthrough SARS-CoV-2 infection; 2 (11%), 12 (63%), and 5 (26%) after the second, third, and fourth vaccine dose, respectively. Based on either genotyping or the timing of the infection (≥ January 2022), all other cases were presumed to be Omicron. Only one patient required oxygen treatment and was therefore admitted for 5 days at the hospital. The majority of SARS-CoV-2-infected patients (64%) received mAB (a single dose of 500 mg of sotrovimab was given to this population) (**Table 1**). Three out of 19 patients who had a sample collected after COVID-19 became anti-N positive.

**Table 1:** Baseline characteristics of 33 study participants with common variable immunodeficiency.

	*n* = 33
**Demographics**	
Male: *n* (%)	14 (42%)
Age: Median (IQR)	49.5 (31.8, 67.7)
Age of CVID symptom onset: Median (IQR)	28.8 (15.2, 46.2)
Age at CVID diagnosis: Median (IQR)	40.5 (22.8, 58.8)
Born in Denmark: *n* (%)	33 (100%)
Meet old ESID CVID criteria: *n* (%)	33 (100%)
Meet revised ESID CVID criteria: *n* (%)	22 (67%)
Revised ESID criteria not met due to naïveT cells <10% and unknown T-cell proliferation: *n* (%)	11 (33%)
Latest IgG: Median (IQR)	6.4 (4.7, 8.3)
**Clinical manifestation**	
Only infection: *n* (%)	13 (39%)
**Immunoglobulin replacement therapy (IgRT)**	
IgRT: *n* (%)	28 (85%)
**Type of IgRT**	
Hizentra: *n* (%)	15 (54%)
HyQvia: *n* (%)	6 (21%)
Privigen: *n* (%)	7 (25%)
**Immunomodulating therapy**	
Current systemic glucocorticoids: *n* (%)	5 (15%)
Previous anti-CD20 mAB: *n* (%)	3 (9%)
**SARS-CoV-2 vaccination**	
0 vaccine dose: *n* (%)	3 (9%)
≥2 vaccine doses: *n* (%)	30 (91%)
≥3 vaccine doses: *n* (%)	28 (85%)
≥4 vaccine doses: *n* (%)	16 (48%)
**Type of SARS-CoV-2 vaccination**	
Pfizer/BioNTech (Comirnaty): *n* (%)	29 (97%)
Moderna (Spikevax): *n* (%)	1 (3%)
**COVID-19**	
COVID-19 infected: *n* (%)	22 (67%)
Treated with monoclonal antibodies: *n* (%)	14 (42%)

CVID, common variable immunodeficiency; ESID, European Society for Immunodeficiency; mAB, monoclonal antibodies.

**Figure 1:**
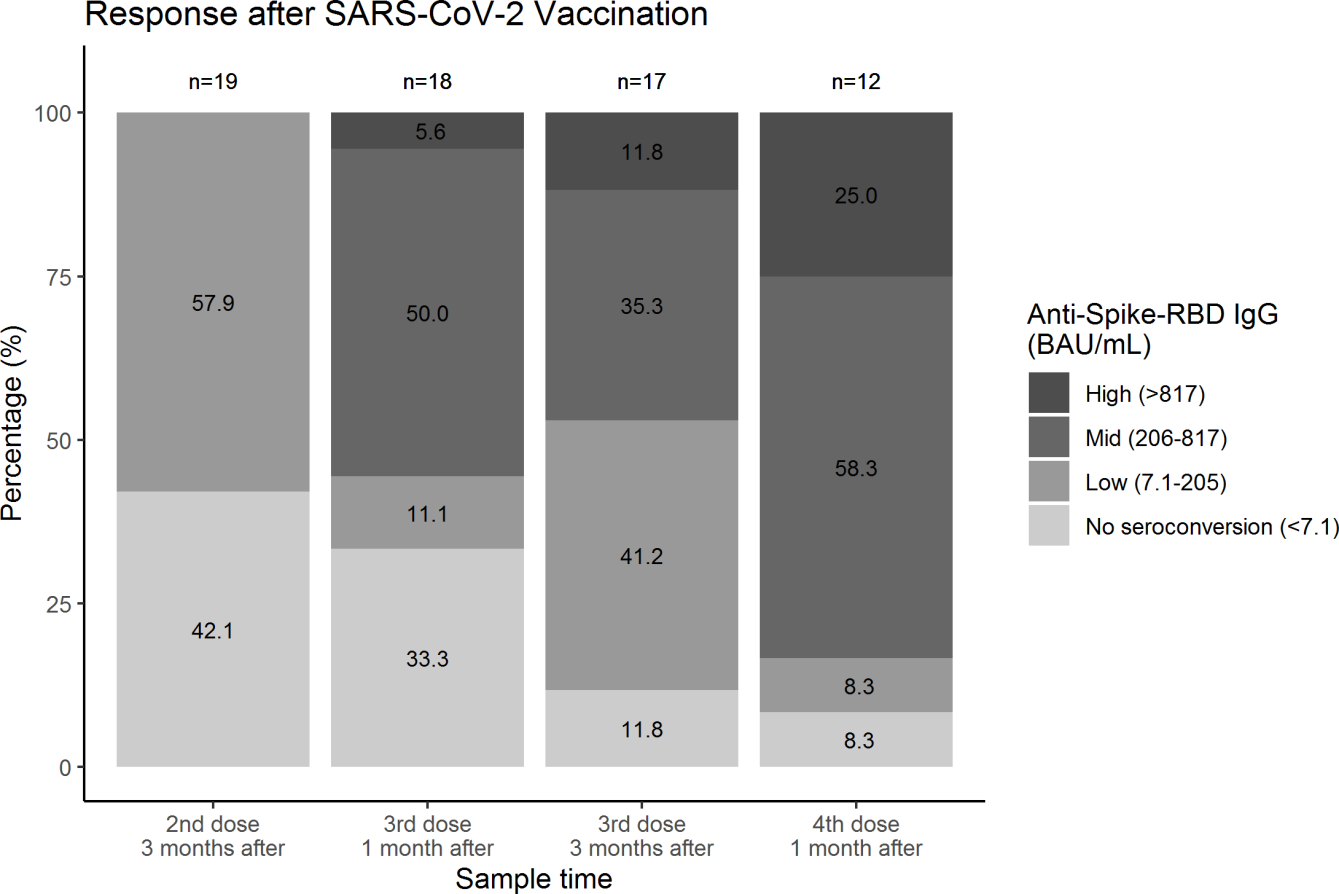
Proportion of individuals with common variable immunodeficiency within each category of anti-spike protein receptor-binding domain IgG antibody response after different doses of SARS-CoV-2 vaccination. Samples were collected from 27 individuals with common variable immunodeficiency. Samples collected after administration of monoclonal antibodies were excluded. Proportions were compared with McNemar; 3 months after the second and third dose p = 0.248 and 1 month after the third and fourth dose p = 0.480. The number of missing samples in each column (from left to right) were n = 8, n = 9, n = 10, and n = 15, respectively. The missing samples were included in **Supplementary Figure 2**. RBD, receptor-binding domain.

With increasing number of vaccinations, the proportion of patients who seroconverted increased non-significantly (**Figure 1**). After the second vaccine, 8 (42%) out of 19 had undetectable antibody levels, and this fell to 2 (12%) out of 17 after the third dose (*p* = 0.248). Similarly, 6 (33%) out of 18 had undetectable antibody levels 1 month after the third dose and this number was only 1 (8%) out of 12 after the fourth dose (*p* = 0.480) (**Figure 1**). A sensitivity analysis, in which the window of the sampling was extended (i.e., 1 month: 0.5–2 months and 3 months: 2.1–6 months) showed similar results (**Supplementary Figure 3**).

**Figure 2:**
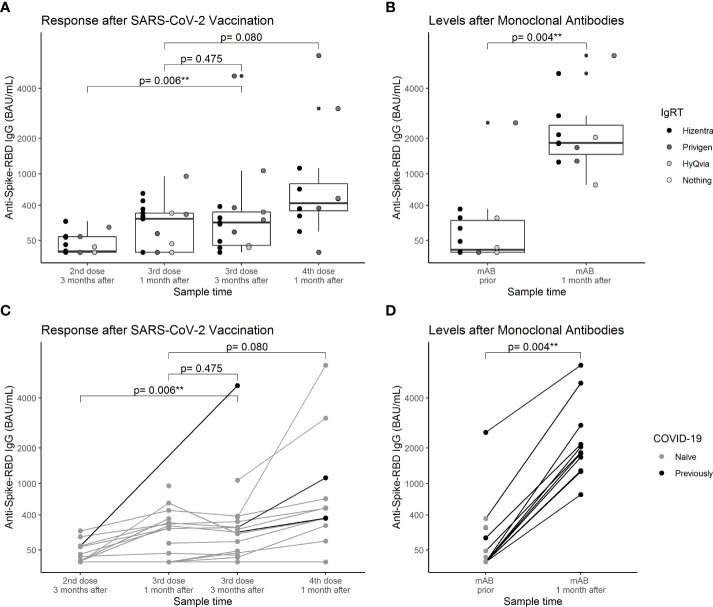
Anti-spike protein receptor-binding domain IgG antibody levels after different doses of SARS-CoV-2 vaccination and treatment with monoclonal antibodies in individuals with common variable immunodeficiency. The anti-spike receptor-binding domain IgG antibody response after SARS-CoV-2 vaccination (left figures, **A** and **C**) and after monoclonal antibody treatment (right figures, **B** and **D**). In the left figures, samples collected after administration of monoclonal antibodies were excluded. Data were compared using paired Wilcoxon sign rank tests. Scatters were colored according to immunoglobulin replacement therapy (upper figures, **A** and **B**) and COVID-19 (previous vs. naïve) at the time of sampling (lower figures, **C** and **D**). One and three months after the third dose and 1 month after the fourth dose, there were no significant variation in spike IgG antibodies across groups of IgRT in Kruskal–Wallis tests (p = 0.881, p = 0.437, and p = 0.460, respectively). RBD, receptor-binding domain; IgRT, immunglobulin replacement therapy. ** p<0.01.

The median levels and dynamics of anti-S-RBD after each dose of the SARS-CoV-2 vaccine in mAB naïve patients are illustrated in **Figure 2** (left column). The median levels were 8.8 BAU/ml (IQR: 7.0; 70 BAU/ml) and 187 BAU/ml (IQR: 27; 308 BAU/ml) 3 months after the second and third dose, respectively. The levels increased by 165 BAU/ml [95% confidence interval (95% CI): 85; 2,280 BAU/ml, *p* = 0.006] between the two doses. When we excluded one patient with previous COVID-19, the increase was 108 BAU/ml (95% CI: 37; 175 BAU/ml, *p* = 0.009). One month after the third and fourth dose, the levels were 226 BAU/ml (IQR: 7.0; 294 BAU/ml) and 433 BAU/ml (IQR: 326; 798 BAU/ml) with the increase being borderline significant (193 BAU/ml, 95% CI: −22; 569 BAU/ml, *p* = 0.080). When two patients with previous COVID-19 were excluded, the increase did not attenuate and was insignificant (185 BAU/ml, 95% CI: −22; 253 BAU/ml, *p* = 0.208). No significant decline was observed between 1 and 3 months after the third dose (−22 BAU/ml, 95% CI: −207; 20 BAU/ml, *p* = 0.475). Prior to mAB therapy, the median level of anti-S-RBD was 26 (IQR: 7.0; 186 BAU/ml) and the median level 1 month after was 2,083 BAU/ml (IQR: 1,815; 3,294 BAU/ml) (**Figure 2**, right column). The elevation in antibody levels was 2,035 BAU/ml (95% CI: 1,372; 3,131 BAU/ml, *p* = 0.004). Those who received the fourth vaccine dose had lower anti-S-RBD levels than those who received mAB without the fourth vaccine dose (−1,502 BAU/ml, 95% CI: −2,466; −570 BAU/ml, *p* = 0.009) 1 month after intervention.

**Table 2** shows the different levels of antibodies in IgRT samples between August 2021 and April 2022. The levels of anti-S-RBD and anti-N varied substantially between products (*p* < 0.001 and *p* < 0.001). When comparing the anti-S-RBD levels of the patients by the different types of IgRT, we found no significant difference between types of IgRT treatment (1 month after the third dose: *p* = 0.881, 3 months after the third dose: *p* = 0.437, and 1 month after the fourth dose: *p* = 0.460). Among three unvaccinated and mAB naïve patients, blood drawn during follow-up showed anti-S-RBD levels below threshold in two of the patients on HyQvia, whereas one Hizentra-treated patient converted to a low level of antibodies (anti-S-RBD: 18 BAU/ml) in December 2021.

**Table 2:** Anti-nucleocapsid IgG and anti-spike receptor-binding domain IgG antibody concentrations in common immunoglobulin replacement therapy products between August 2021 and April 2022.

Product		Nucleocapsid IgG antibodies (BAU/ml)		Spike-RBD IgG antibodies (BAU/ml)	
	*n*	Median	IQR	Median	IQR
Hizentra	3	5.6	(4.0; 5.9)	1086	(587; 1,266)
Privigen	32	0	(0; 1.5)	14	(7.3; 19)
HyQvia	13	0	(0; 0)	3.2	(2.9; 5.0)
Octagam	8	4.3	(3.0; 4.5)	379	(240; 884)

Patients who received oral glucocorticoid therapy or previous anti-CD20 therapy had lower levels of anti-S-RBD, but this was not significant compared to patients who had not received this therapy (*p* = 0.197 and *p* = 0.050) after the second dose. Similar results were found after the third (*p* = 0.175 and *p* = 0.226) and fourth dose (*p* = 0.192 and *p* = 0.111). However, the number of patients in these analyses was small. Patients presenting with infections only compared to patients presenting with autoimmune/granulomatous/lymphoproliferative disease had a significantly higher anti-S-RBD response after the second dose (*p* = 0.043), but not after the third (*p* = 0.509) and fourth dose (*p* = 0.522). Patients fulfilling the revised ESID criteria had a higher response after the second dose compared to those with unknown T-cell proliferation and naïve T cells <10% (*p* = 0.023).

## Discussion

This study shows that a third SARS-CoV-2 vaccination can increase the antibody levels in CVID patients, and the fourth dose tend to have the same effect. In addition, most of the two-dose non-responders seroconverted by repeating immunization. Still, higher levels of anti-S-RBD were observed after mAB treatment compared to vaccination. The levels of antibodies in IgRT products varied greatly, but the IgRT treatment did not seem to affect the anti-S-RBD levels in our CVID patients. To our knowledge, this is the first study to examine the antibody levels after a third and fourth SARS-CoV-2 vaccination and after mAB treatment in CVID patients.

Different reports have demonstrated rather good vaccination responses with detectable humoral and cellular responses in up to 80% of CVID patients after the second vaccination (7, 8, 11, 21, 22). At the same time, both B- and T-cell responses of CVID patients were inferior to healthy controls (11), and the neutralizing capacity seemed low (8%–50%) (11, 21). Although the affinity was not assessed in this study, our study indicates that repeated vaccination might be beneficial in CVID, as antibody levels increased.

SARS-CoV-2 antibody cutoff levels for protection against breakthrough COVID-19 have not been possible to establish (23). Furthermore, the vaccine response seems highly variable among CVID patients. Hence, it is difficult to agree on the optimal number of vaccine doses in CVID. It has been reported that mRNA-based vaccines induce less humoral protection against Omicron compared to the wild-type strains (24). This could explain the high breakthrough infection rate in this study during the Omicron period, despite multiple vaccine doses. Fortunately, protective T-cell response might be long-lasting (25) and robust to new mutations in virus strains (23), which is especially important in people with isolated B-cell deficiencies (26). We cannot rule out that the fairly mild COVID-19 cases in this study were primarily due to a low pathogenic strain and/or mAB treatment (27). Still, it seems that our patients were sufficiently protected against severe disease.

It is not clear whether the four-dose vaccination strategy is sufficient to protect against severe COVID-19, as the oldest and most ill CVID patients with COVID-19 received mAB. Still, with the emerging viral variants, resistance may evolve towards the different mAb, even when used as a two-antibody cocktail that bind to epitopes that overlap with the rapidly evolving receptor binding motif (RBM), why the effect of mAB may quickly change (28). Furthermore, mAB will not stimulate a T-cell response, are more costly than vaccines, and despite a median half-life of approximately 49 days for, e.g., sotrovimab (29), the overall duration of effectiveness is not yet fully known in CVID patients, which support the use of vaccines. Hence, further attempts should be made to evaluate the importance of mAB as pre- and post-exposure prophylaxis in CVID.

During the pandemic, companies producing IgRT, such as Octapharma^®^ and Takeda^®^, have reported rapidly increasing SARS-CoV-2 antibodies in commercial lots from pooled donated plasma (4, 5). We showed high variation in the different IgRT products, with some products containing very low concentrations why a clinical effect seems unlikely. Moreover, it takes at least 5–6 months to prepare a ready-to-use IgRT product from donated plasma (4, 5). Hence, products from different time periods (i.e., with high or low transmission and vaccination rate), may explain some of the variation in the product levels of antibodies. Although some IgRT products might provide small protection, the processing delay means that the protection against new strains are limited, and IgRT should probably be supplemented with other SARS-CoV-2 protective interventions.

This study has some limitations. The response after the fourth dose might have been confounded by COVID-19 between the third and fourth dose; nevertheless, the amplitude of the response was unchanged when excluding these patients. Another limitation of this study is that we did not measure the T-cell response and the neutralizing effect of the antibodies, but a study has been set up to examine this further. Moreover, this was a retrospective study with real-life data; hence, we had missing antibody samples from some patients and the time of sampling varied. As of the half-life of sotrovimab, the amplitude might have been somewhat affected by smaller differences in timing of the blood test. However, as the levels of anti-S-RBD observed after mAB were substantially higher compared to a fourth vaccine, we do not believe that this has had a major impact on the overall estimates. Finally, the small sample size limits the power and sub-analyses of this study. Nevertheless, to our knowledge, this study is currently the only study within the field of the antibody response in CVID patients after the third and fourth SARS-CoV-2 vaccination.

In conclusion, the antibody response in CVID patients increased after the third dose of SARS-CoV-2 vaccine and the fourth dose seemed to have a similar effect. Furthermore, some of the initial non-responders seroconverted after further doses. Compared to four doses of vaccines, mAB provided higher antibody levels, but as mAB is a passive immunization, mABs are unable to stimulate memory and other pathways in the immune response. Nonetheless, in individuals with a very low vaccination response or no seroconversion, prophylactic mAB could be an efficient alternative or supplement to vaccines. Some IgRT products had measurable levels of SARS-CoV-2 antibodies; however, it seems unlikely that IgRT currently serves as a sufficient COVID-19 prophylaxis in CVID.

## Data Availability Statement

The original contributions presented in the study are included in the article/**supplementary material**, further inquiries can be directed to line.dahlerup.rasmussen@rsyd.dk.

## Ethics Statement

Ethical review and approval was not required for the study on human participants in accordance with the local legislation and institutional requirements. The patients/participants provided their written informed consent to participate in this study.

## Author Contributions

Conception and design: LR IJ, AH, and AN. Collection and assembly of data: AH, AN, and LR. Analysis of the data: BN and AN. Statistical expertise: BN and LR. Interpretation of the data: BN, CD, AN, and LR. Drafting of the article: CD, BN, and LR. Critical revision of the article for important intellectual content: BN, CD, MB, IJ, AH, AN, and LR. Final approval of the article: BN, CD, MB, IJ, AH, AN, and LR.

## Acknowledgments

We are grateful for the support from the staff at the Department of Infectious Diseases at Odense University Hospital (OUH), Southern Denmark. A special thanks goes to the following nurses in the outpatient department: Louise Trier Hansen and Mette Bjerg Friis.

## Conflict of Interest

LR has after submission of the paper received support concerning conference fee and travelling by Takeda for the coming ESID conference. CD has unrelated to the current study received research grants from Takeda. MB has received consulting honorariums from Janssen and Kite/Gilead, in areas unrelated to this research.

The remaining authors declare that the research was conducted in the absence of any commercial or financial relationships that could be construed as a potential conflict of interest.

## Publisher’s Note

All claims expressed in this article are solely those of the authors and do not necessarily represent those of their affiliated organizations, or those of the publisher, the editors and the reviewers. Any product that may be evaluated in this article, or claim that may be made by its manufacturer, is not guaranteed or endorsed by the publisher.

## Supplementary Material

Supplementary Figure 1 Flow chart of study participants. Patients were able to contribute samples in both the vaccine study and in the monoclonal antibody study. CVID, common variable immunodeficiency; mAB, monoclonal antibody.

Supplementary Figure 2 Proportion of individuals with common variable immunodeficiency within each category of anti-spike protein receptor-binding domain IgG antibody response after different doses of SARS-CoV-2 vaccination. Samples were collected from 27 individuals with common variable immunodeficiency. Samples collected after administration of monoclonal antibodies were excluded.

Supplementary Figure 3 Proportion of individuals with common variable immunodeficiency within each category of anti-spike protein receptor-binding domain IgG antibody response after different doses of SARS-CoV-2 vaccination with extended windows* for sampling. Samples were collected from 29 individuals with common variable immunodeficiency. Samples collected after administration of monoclonal antibodies were excluded. *Extended windows for sampling: 1 month (0.5 – 2 months) and 3 months (2.1-6 months). RBD, receptor-binding domain.

## Abbreviations

SARS-CoV-2, Severe acute respiratory syndrome coronavirus-2; COVID-19, coronavirus disease; PID, primary immune deficiency; CVID, common variable immunodeficiency; IgRT, immunoglobulin replacement therapy; mAB, monoclonal antibodies; anti-S-RBD, SARS-CoV-2 spike receptor-binding domain IgG antibody; anti-N, SARS-CoV-2 nucleocapsid IgG antibodies; OUH, Odense University Hospital; IQR, interquartile ranges; 95% CI, 95% confidence interval.
