# Supplementary figures and images for: Antibody response following the third and fourth SARS-CoV-2 vaccine dose in individuals with common variable immunodeficiency
Source: Front Immunol. 2022 Jul 28;13:934476. doi: 10.3389/fimmu.2022.934476 (PMC9366053; doi:10.3389/fimmu.2022.934476)

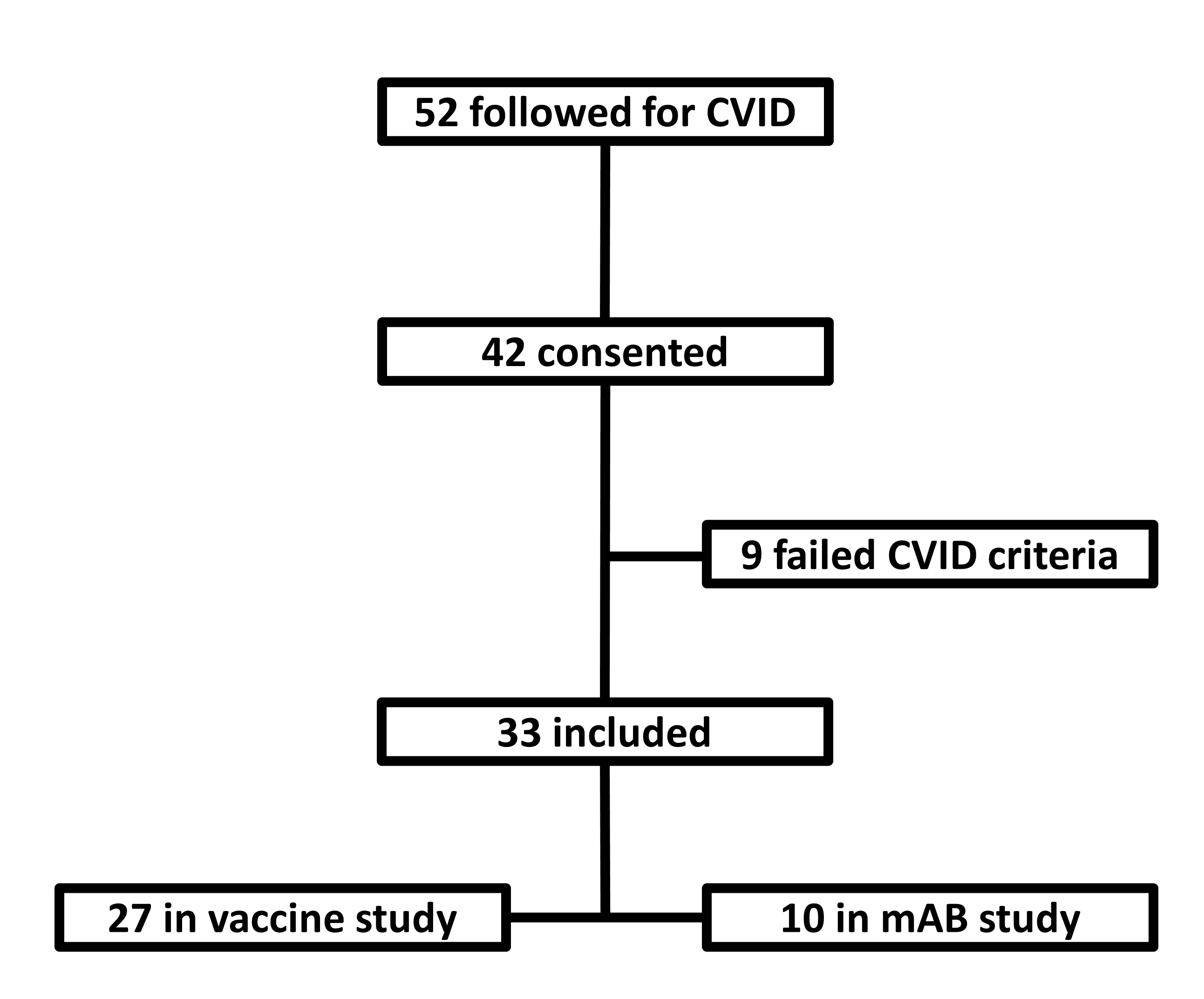

Supplement: Supplementary Figure 1 — Flow chart of study participants. Patients were able to contribute samples in both the vaccine study and in the monoclonal antibody study. CVID, common variable immunodeficiency; mAB, monoclonal antibody. [file Image_1.tif]

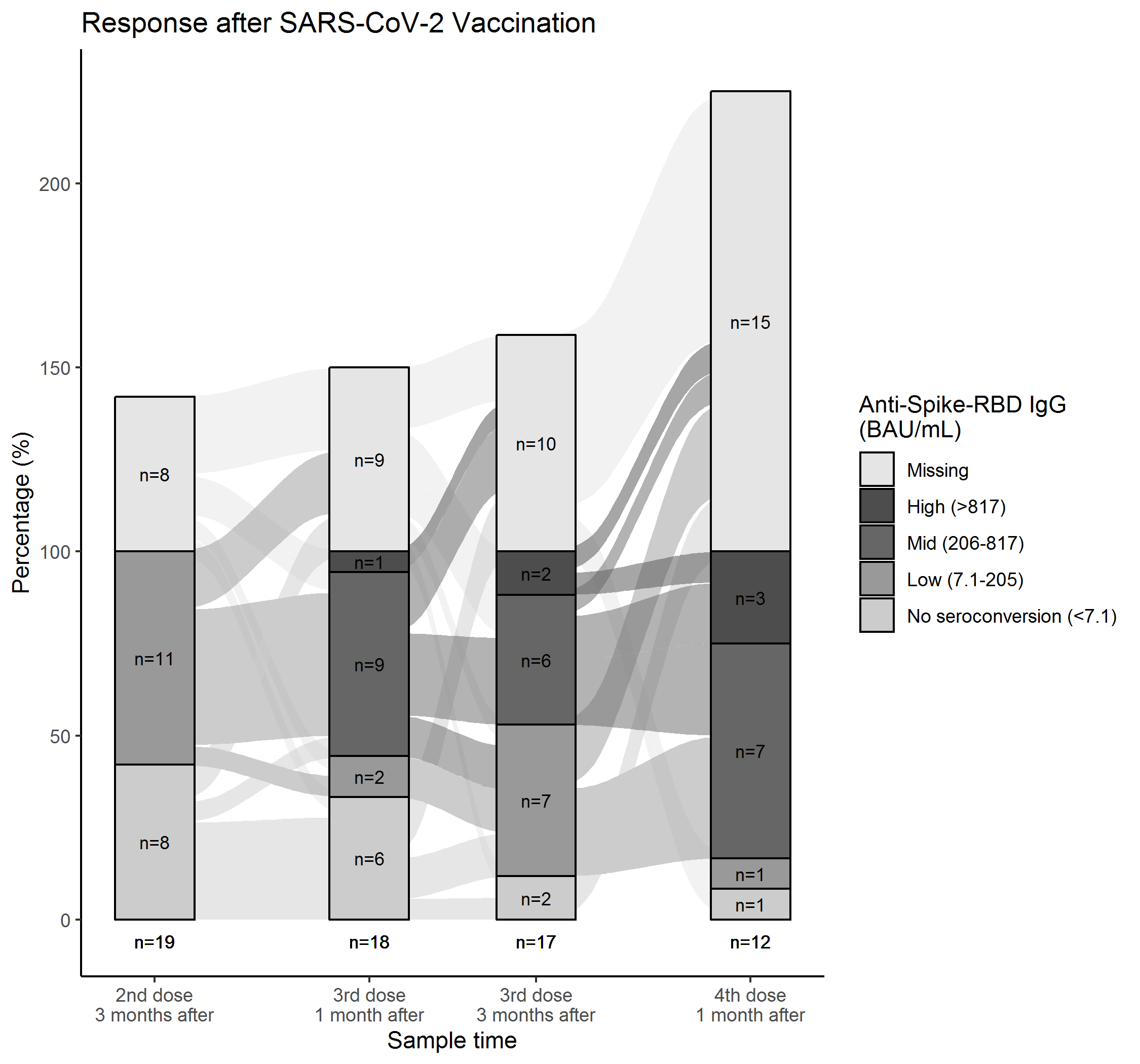

Supplement: Supplementary Figure 2 — Proportion of individuals with common variable immunodeficiency within each category of anti-spike protein receptor-binding domain IgG antibody response after different doses of SARS-CoV-2 vaccination. Samples were collected from 27 individuals with common variable immunodeficiency. Samples collected after administration of monoclonal antibodies were excluded. [file Image_2.tiff]

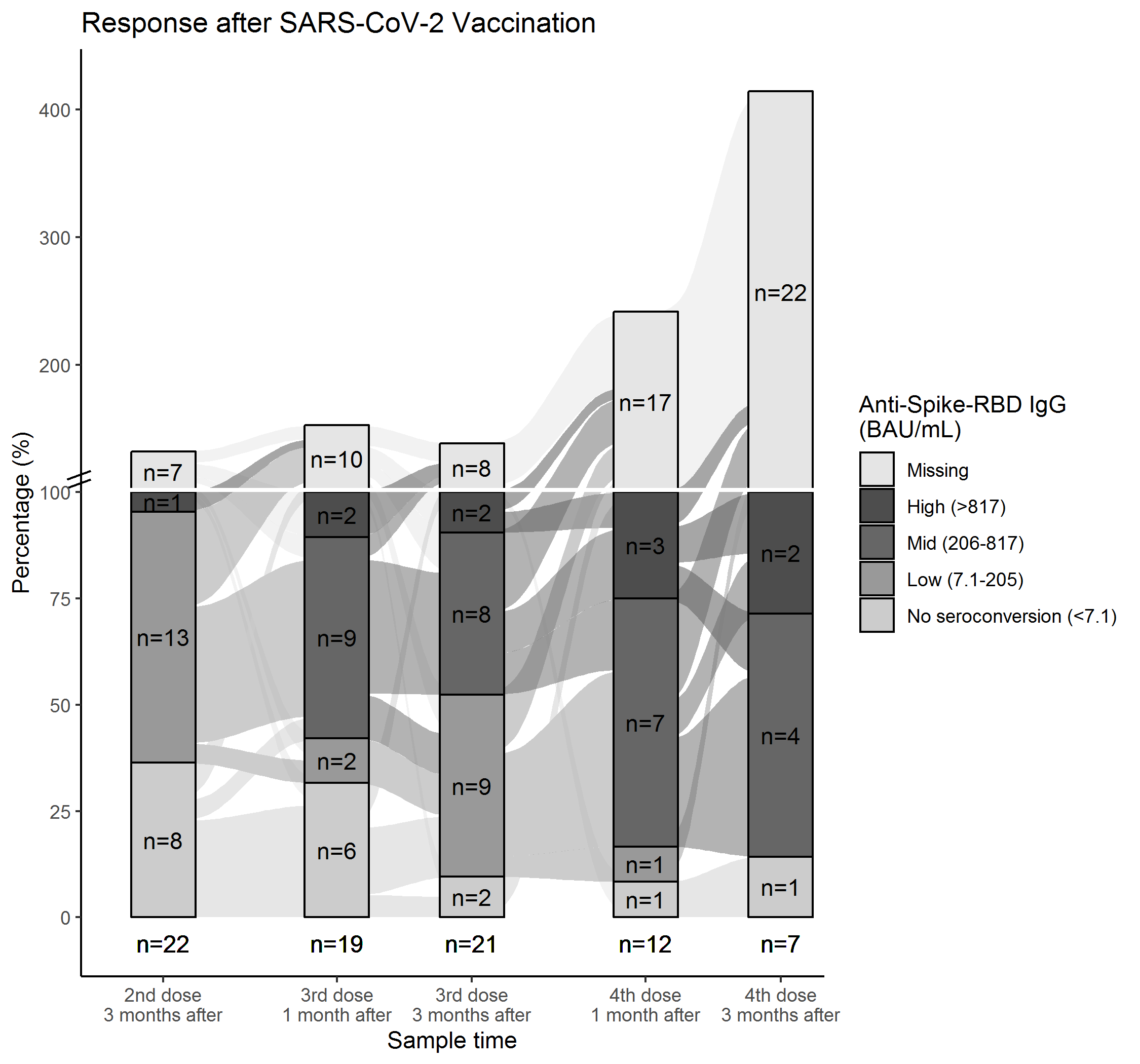

Supplement: Supplementary Figure 3 — Proportion of individuals with common variable immunodeficiency within each category of anti-spike protein receptor-binding domain IgG antibody response after different doses of SARS-CoV-2 vaccination with extended windows* for sampling. Samples were collected from 29 individuals with common variable immunodeficiency. Samples collected after administration of monoclonal antibodies were excluded. *Extended windows for sampling: 1 month (0.5 – 2 months) and 3 months (2.1-6 months). RBD, receptor-binding domain. [file Image_3.tiff]
